# ACM: Accuracy-Aware Collaborative Monitoring for Software-Defined Network-Wide Measurement

**DOI:** 10.3390/s22207932

**Published:** 2022-10-18

**Authors:** Jiqing Gu, Chao Song, Haipeng Dai, Lei Shi, Jinqiu Wu, Li Lu

**Affiliations:** 1 School of Computer Science and Engineering, University of Electronic Science and Technology of China, Qingshuihe Campus, Chengdu 611731, China; 2 Department of Computer Science and Technology, Nanjing University, Nanjing 210023, China

**Keywords:** Count–Min sketch, large flows, software-defined network-wide measurement, collaborative monitoring

## Abstract

Software-defined measurement (SDM) is a simple and efficient way to deploy measurement tasks and collect measurement data. With SDM, it is convenient for operators to implement fine-grained network-wide measurements at the flow level, from which many important functions can benefit. The prior work provides mechanisms to distribute flows to monitors, such that each monitor can identify its non-overlapped subset of flows to measure, and a certain global performance criterion is optimized, such as load balance or flow coverage. Many applications of network management can benefit from a function that can find large flows efficiently, such as congestion control by dynamically scheduling large flows, caching of forwarding table entries, and network capacity planning. However, the current network-wide measurements neglect the diversity of different flows as they treat large flows and small flows equally. In this paper, we present a mechanism of accuracy-aware collaborative monitoring (ACM) to improve the measurement accuracies of large flows in network-wide measurements at the flow level. The structure of the sketch is an approximate counting algorithm, and a high-measurement accuracy can be achieved by merging the results from multiple monitors with sketches, which is termed as collaborative monitoring. The core idea of our method is to allocate more monitors to large flows and achieve the load balance to provide accuracy-aware monitoring. We modeled our problem as an integer–linear programming problem, which is NP-hard. Thus, we propose an approximation algorithm, named the improved longest processing time algorithm (iLPTA); we proved that its approximation ratio is $\left(\frac{1}{2}+\frac{n}{l}\right)$. We propose a two-stage online distribution algorithm (TODA). Moreover, we proved that its approximation ratio is $\left(1+\frac{n}{l\;-\;1}\right)$. The iLPTA is an offline approximation algorithm used to assign monitors for each flow, which prove the validity and feasibility of the core idea. The TODA is an online algorithm that attempts to achieve the load balance by selecting the monitor with the smallest load to a large flow. Our extensional experiment results verify the effectiveness of our proposed algorithms.

## 1. Introduction

Software-defined measurement (SDM) is an efficient and simple way to deploy measurement tasks and collect measurement data [1,2,3]. It employs a programmable data-plane based on commodity switches, and a flexible control plane so that the operators can implement variable measurement algorithms easily. It provides real-time visibility into traffic in enterprises and data center networks by permitting largely instantiated network-wide measurement tasks. With SDM, it is convenient for operators to implement fine-grained network-wide measurements at the flow level, from which many important functions can benefit. Network operators routinely collect flow-level network-wide measurements to guide several network management applications [4,5,6].

**Figure 1:**
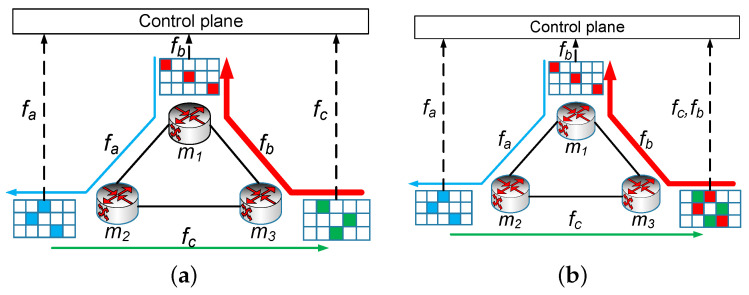
An example of network-wide measurements under a triangle topology. (**a**) Non-overlapped monitoring; (**b**) Collaborative monitoring.

The flow-level network-wide traffic measurements are based on the association between the flow and monitors, which is called flow distribution [7]. The existing studies on network measurements are mainly divided into two categories. The first category focuses on designing compact and efficient data structures that can significantly improve measurement accuracy or throughput [8,9,10,11,12]. However, these research studies mainly focus on single monitors and do not consider network-wide measurements. While the second one focuses on the flow distribution for network-wide traffic measurements. Many studies [4,7,13,14] provide mechanisms to distribute flows to monitors such that each monitor can identify its non-overlapped subset of flows to measure; a certain global performance criterion is optimized such as load balance and flow coverage which we term as non-overlapped monitoring. We take an example of network-wide measurements under a triangle topology with three monitors, as shown in Figure 1a. There are three flows, which are one large flow, $f_{b}$, and two small flows, $f_{a}$ and $f_{c}$. Each flow is measured only once by a monitor, and we call this kind of flow distribution ’non-overlapped monitoring’.

It is well known in real network traffic that the distribution of flow size (the number of packets in a flow) follows Zipf distribution [10], i.e., the majority are small flows, while the minority are large flows. Many applications of network management benefit from a function that can find large flows efficiently and timely, such as congestion control by dynamically scheduling large flows [15], network capacity planning [5], anomaly detection [16], and caching of forwarding table entries [17]. However, the current work neglects the diversity of different flows as they treat large flows and small flows equally. Traditional flow-level measurement with non-overlapped monitoring will distribute these flows to monitors uniformly, one flow per monitor as shown in Figure 1a. Because large flow $f_{b}$ is measured on only one Count–Min sketch with a fixed size, the estimation accuracy of large flow cannot be improved no matter which monitor it is distributed to. However, large flows are more important than small flows for network operators [5,15,16,17]. Thus, accuracy-aware measurements for large flows are vital where the estimation accuracies of large flows can be guaranteed.

Many literature studies [8,9,10,18] insist that sketch data structures can give an inaccurate count for the large flows, on which administrators must focus. The accuracy of estimating one flow can be improved by merging the results if it is measured on several monitors. Take the Count–Min sketch [19] as an example, merging two sketches with the same size can be regarded as a bigger Count–Min sketch with double height. We term this as collaborative monitoring to improve the measuring accuracy by merging the results from multiple monitors with sketches. As shown in Figure 1b, $f_{b}$ is measured on monitor $m_{1}$ and monitor $m_{3}$, so the accuracy of estimating $f_{b}$ is improved.

In this paper, we propose a mechanism of accuracy-aware collaborative monitoring (ACM). The basic idea is for small flows, only one monitor is assigned for measurement, but for large flows, multiple monitors are distributed by collaborative monitoring. The challenges of designing accuracy-aware collaborative monitoring are two folds. In order to avoid the overload of monitors, it is necessary to perform load balance. Thus, how to ensure load balance under accuracy-aware collaborative monitoring is the first challenge. Since the flow size is not known in advance, it is a challenge to provide an online algorithm without awareness of flow size. We summarize our contributions as follows:

- We propose a mechanism of Accuracy-aware Collaborative Monitoring (ACM) for software-defined network-wide measurements to make full use of monitor resources, and improve the estimation accuracy for large flows.
- We translate the problem of network-wide measurements into a two-stage load-balance problem. We propose an approximation algorithm named improved longest processing time algorithm (iLPTA), and prove that its approximation ratio is $\left(\frac{1}{2}+\frac{n}{l}\right)$.
- We provide a two-stage online distribution algorithm (TODA) to adapt the actual network environment. Then we prove that its approximation ratio is $\left(1+\frac{n}{l-1}\right)$.

The rest of this paper is organized as follows. Section 2 surveys the related work; Section 3 introduces the problem of network-wide measurement; Section 4 discusses collaborative monitoring; Section 5 presents an approximation algorithm and an online algorithm; Section 6 evaluates the performance of the proposed approach; and the last section concludes this paper and discusses future work.

## 2. Related Work

In this section, we present the related work on three topics, i.e., flow-level network-wide measurement, sketch for measurement, and large flows.

Recently, well-known network-wide measurements systems for flow distribution include CSAMP [4], DCM [13], LEISURE [14] and NSPA [7]. Sekar et al. in [4] present CSAMP, a system that takes a network-wide approach to flow monitoring, which provides higher flow coverage, achieves the goals of fine-grained network-wide flow coverage, and efficiently leverages available monitoring capacity. Yu et al. in [13] propose a Distributed Collaborative Monitoring (DCM) system for SDN-enabled flow monitoring and measurement, which use two-stage Bloom filters as the DCM data plane to represent monitoring rules in an efficient and reliable way. Chang et al. in [14] present a centralized optimization framework, LEISURE (Load-Equalized measurement), for load-balance network measurement workloads across distributed monitors. Xu et al. in [7] propose a new lightweight solution to the flow distribution problem. By minimizing data-plane space and processing overhead, it follows the design principle of alleviating the data-plane complexity.

There are many research studies on sketches for traffic measurement in the research community, which offer efficient measurement support for individual management tasks. Sketches can be used for many measurement tasks, such as heavy hitters detection [20], traffic change detection [21], flow size estimation [19], and DDoS detection [1]. For example, heavy hitters are large flows that spend more than a fraction *T* of the link capacity during a time interval. To recognize heavy hitters, we first use a Count–Min sketch [19] to maintain the counts for each flow. Then, we recognize potential flows that hashed to heavy counters in a reversible sketch [22], and verify their actual count using the Count–Min sketch.

Many researchers have analyzed the problem of large flows in network measurements. The network data stream follows the Zipf distribution, i.e., 20% of the top-ranked flows account for more than 80% of total traffic. Roy et al. in [9] believed that sketch data structures could give an inaccurate count for the most frequent items. Therefore, the authors proposed augmented sketch (ASketch), based on a pre-filtering stage that dynamically identifies and aggregates the most frequent items. Similar to the augmented sketch, Zhou et al. in [8] proposed a meta-framework called cold filter (CF), which enables faster and more accurate stream processing. Unlike the existing filters, which mainly focus on hot items, its filter captures cold items in the first stage and hot items in the second stage, respectively. Yang et al. in [10] applied a novel strategy, called count-with-exponential-decay, to accomplish the space–accuracy balance by removing small flows through decaying actively; this strategy minimizes the impact on large flows and achieves high precision in finding top-*k* elephant flows. The work [23] used a sampling method in monitors, which collected flow-level statistics of the selected flows. There are also some works [24,25] about flow monitoring in the control plane, which is not in the scope of this paper. However, these studies are limited to a single monitor and do not consider network-wide measurements.

Our work differs from the aforesaid studies in that we improve the estimation accuracies of large flows in network-wide measurements by collaborative monitoring.

## 3. Problem of Network-Wide Measurement

In this section, we introduce the system framework of software-defined network-wide measurements and present the Count–Min sketch as an example of the monitor. Then, we analyze the problem of network-wide measurements at the flow level. All the notations used in this paper are listed in Table 1.

### 3.1. System Overview

**Figure 2:**
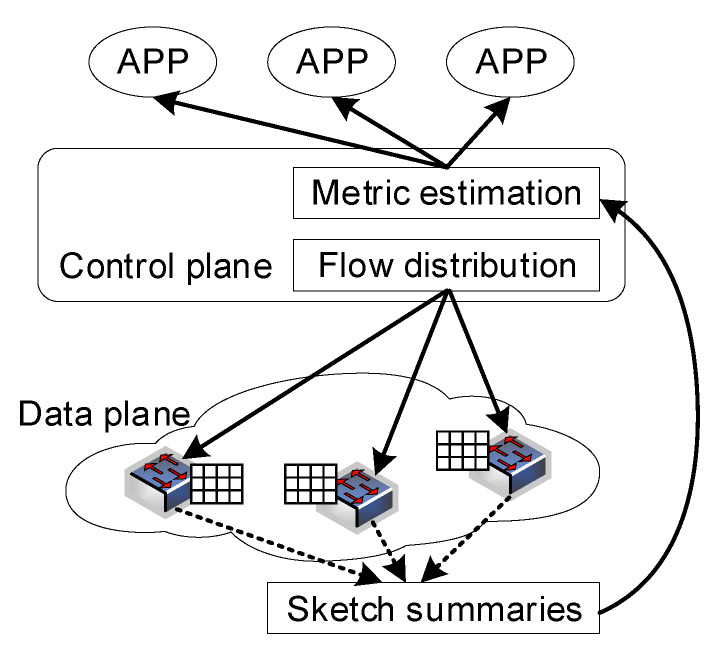
Framework of software-defined network-wide measurements.

For network-wide measurement tasks, many studies [3,12,21] have investigated software-defined measurements with network-wide sketches. Figure 2 shows a high-level overview of software-defined network-wide measurements. In the data plane, the switches or routers on which sketches are deployed in the data-plane component are called monitors. We used the monitor to briefly represent sketches and the underlying switch or router in the rest of this paper. The APPs provide services for operators or talents, such as DDoS detection and heavy hitter detection. Based on the requirements of APPs, the control plane needs to decompose services into basic measurement tasks at the flow level, and decide how to distribute measurement tasks to monitors, which is called flow distribution [7]. Then the monitors perform the monitoring operations and report sketch summaries to the control plane. Finally, the metric estimation module in the control plane calculates application-specific data, such as top-*k* flows, and reports the results to the APPs [21]. We assume the controller has a powerful processing capacity and throughput.

In order to demonstrate the basic idea of the sketch, we took a Count–Min sketch [19] as an example to illustrate how it works. The data structure consists of a two-dimensional array with w×h cells of width *w* and height *h*. Each hash function corresponds to one *h* 1-dimensional array with *w* cells, and it is utilized to approximately maintain the frequency counts of a large number of distinct flows in a data stream. We employed *h* pairwise independent hash functions, where each function mapped onto the uniform random integers in the range [0,1,2⋯w]. To recover the size of a given flow, we determined the set of *h* cells onto which each *h* hash function mapped, and computed the minimum value among all these cells. Let $a_{i}$ denote the real value of the count being estimated for flow $f_{i}$. The estimated count is at least equal to $a_{i}$ since we are addressing non-negative counts only, and it may be over-estimated because of collisions among hash cells. The result is that a probabilistic upper-bound to the estimation can be determined [19]. For a data stream with *N* as the sum of the counts of the items received up to now, the estimated count ${\hat{a}}_{i}$ is at most $a_{i}+\frac{e}{w}N$ with a probability of at least $1-e^{-h}$, which is shown as follows:

(1) $$P\left\{{\hat{a}}_{i}\leq a_{i}+\frac{e}{w}N\right\}\geq 1-e^{-h}$$

where *e* is the base of the natural logarithm.

For a flow-level network-wide measurement, let G(M,E) represent the network topology, where M denotes the set of *n* monitors and E denotes the set of directed links. A Count–Min sketch with w×h cells of width *w* and height *h* was deployed on each monitor $m_{i}\;\left(i=1,2\cdots n\right)$. Network traffic was modeled as the flow. Each flow was composed of packets that shared a common flow identifier, consisting of several selected fields from the packet header. Let $f_{i}\in F$(i=1,2,⋯,t) denote the *i*th flow with flow size $a_{i}$. The routing path of $f_{i}$ was denoted by $P_{f_{i}}$, which is a subset of monitors. In order to reduce the probability of the hash collision on each Count–Min sketch, $C_{m_{i}}\;\left(i=1,2\cdots n\right)$ was introduced to denote the number of flows that monitored $m_{i}$, which can measure at most in a given measurement interval. We introduce a flow–monitor mapping matrix *D* with *t* rows and *n* columns. The element $d_{ij}$ in the *i*th row and the *j*th column is equal to 1 if the flow $f_{i}$ is measured on monitor $m_{j}$, otherwise, it is equal to 0.

The goal of flow monitoring is to estimate the flow size of each flow efficiently (and nearly exactly) based on the sketch while considering the resource constraints of the switch in a given measurement interval. This paper only focuses on flow monitoring, and the superior functions of a network-wide measurement, such as heavy hitter detection [20] and DDoS detection [1], are out of the scope of this paper.

### 3.2. Accuracy-Aware Network-Wide Measurement

It is well known that the distribution of flow sizes (the number of packets in a flow) follows Zipf distribution [10] in real network traffic. Administrators pay more attention to large flows than small flows because the large flow is related to a variety of tasks, such as load balance and heavy hitter detection. Thus, it is essential to estimate the frequency of the large flows as accurately as possible [8,9].

Many research studies [8,9,10] discuss the importance of large flows since large flows are useful for many applications [5,15,16,17]. However, the current studies [4,7,13,14] in flow-level network-wide measurements hardly pay attention to large flows since they treat large flows and small flows equally, and do not take the measurement accuracy as the evaluation index. As shown in Figure 1a, we gave a simple example of network-wide measurements based on the Count–Min sketch to show the shortages. Even the goal of load balance in network-wide measurements was achieved. That is, one flow per monitor, the estimation accuracy of large flow $f_{b}$ could not be improved no matter which monitor it was distributed to because the flow $f_{b}$ was measured on only one Count–Min sketch with a fixed size. However, operators hope to estimate large flows as accurately as possible. Therefore, the estimation accuracies of large flows may not meet the requirement of network operators.

**Figure 3:**
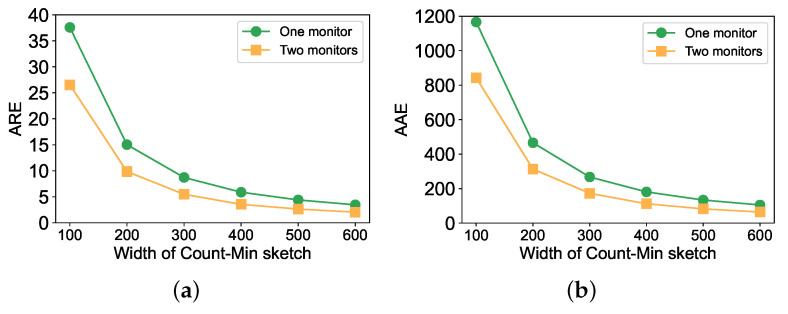
The performances of large flows assigned one monitor and two monitors. (**a**) Performance on ARE; (**b**) Performance on AAE.

We took a set of experiments to verify the measurement with two monitors, which was more accurate than that with one monitor. In the experiment, we selected typical and practical topologies for data center networks and fat-tree topology. We chose fat-tree topology containing 36 monitors (including 16 edge switches, 16 aggregation switches, and 4 core switches). The network traffic data contained 149,769 flows and 2,470,986 packets. We deployed a filter and a Count–Min sketch on each monitor, and performed two methods to evaluate the accuracy of flow counts. One was to measure each flow with a monitor; the other was with two monitors. Figure 3 shows the performances on ARE and AAE of large flows assigned one monitor and two monitors. Both Figure 3a,b show that the measurements of large flows with two monitors are more accurate than with one flow, with the width of the Count–Min sketch increasing.

Thus, we propose accuracy-aware network-wide measurements to improve the accuracy of the subset of flows, which is important in network-wide measurements.

## 4. Collaborative Monitoring

In this section, we analyze the impact of merging multiple sketches on the estimation accuracy. Then, we introduce our method of collaborative monitoring to improve the measuring accuracy and discuss its challenges.

### 4.1. Merging Sketches

**Figure 4:**
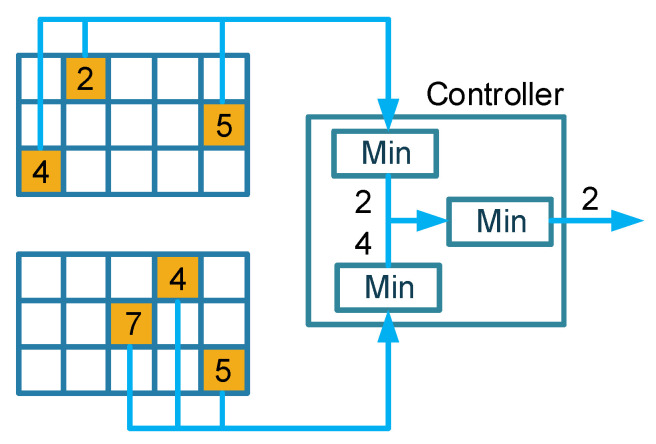
Merge two Count–Min sketches.

We took two Count–Min sketches as an example to illustrate how they were merged. As shown in Figure 4, a flow was measured on two Count–Min sketches with a size of 3×5. This flow was mapped onto six cells indicated by the same colors by six pairwise independent hash functions. When we queried the frequency of this flow, the query result of each sketch was 2 and 4, respectively, by choosing the minimum value of 3 cells. Then we chose the minimum value 2 of the results as merging results of this flow. Because the hash functions of a single Count–Min sketch were independent and the hash functions between two Count–Min sketches were also independent of each other, the noises of six cells were independent of each other. Two Count–Min sketches with size 3×5 can form a bigger Count–Min sketch with size 6×5, which significantly improves the accuracy of the estimation. Thus, it is as if this flow is measured on a bigger Count–Min sketch with double height and the estimation accuracy will be improved according to Equation (1).

Next, we analyzed the impact of merging multiple Count–Min sketches on the estimation accuracy of the general flow. Suppose $f_{i}$ is measured on all monitors on its routing path with the estimated value ${\hat{a}}_{i}^{m}$$\left(m\in P_{f_{i}}\right)$, then the final estimated value of $a_{i}$ is ${\hat{a}}_{i}=\min_{m\in P_{f_{i}}}\left\{{\hat{a}}_{i}^{m}\right\}$.

In the following theorem, we prove a probabilistic bound of multiple Count–Min sketches through merging, similar to the counterpart of the single Count–Min sketch.

**Theorem** **1.** *The estimated value ${\hat{a}}_{i}$ has the following guarantees: ${\hat{a}}_{i}\geq a_{i}$; and, with a probability of at least $1-\delta^{p}$$\left(p=\|P_{f_{i}}{\|}_{1}\right)$,*

(2) $${\hat{a}}_{i}\leq a_{i}+\varepsilon \max_{m\in P_{f_{i}}}{\left\|a^{m}\right\|}_{1}.$$

**Proof.**  For a single Count–Min sketch, the estimated value ${\hat{a}}_{i}^{m}$ has the following guarantees: ${\hat{a}}_{i}^{m}\geq a_{i}$, with the probability of at most δ,

(3) $${\hat{a}}_{i}^{m}\geq a_{i}+\varepsilon {\left\|a^{m}\right\|}_{1}.$$

Then we can derive

(4) $$\begin{array}{cc} \;P\{{\hat{a}}_{i}\geq a_{i}+\varepsilon \max_{m\in P_{f_{i}}}\|a^{m}{\|}_{1}\} & =P\{\forall m,{\hat{a}}_{i}^{m}\geq a_{i}+\varepsilon \max_{m\in P_{f_{i}}}\|a^{m}{\|}_{1}\}\\ & \geq P\{\forall m,{\hat{a}}_{i}^{m}\geq a_{i}+\varepsilon \|a^{m}{\|}_{1}\}\\ & =\delta^{p}. \end{array}$$

   □

We can obtain the following points by comparing them with Equation (1).

1. The probability of multiple Count–Min sketches through merging decreases exponentially, from original δ to $\delta^{p}$, where *p* is the number of Count–Min sketches involved in measuring $f_{i}$.
2. The error range of multiple Count–Min sketches increases, from $\varepsilon \|a^{m_{j}}{\|}_{1}$ to $\varepsilon \max_{m\in P_{f_{i}}}{\left\|a^{m}\right\|}_{1}$. $\varepsilon \max_{m\in P_{f_{i}}}{\left\|a^{m}\right\|}_{1}$ represents the max total traffic of monitors that $f_{i}$ is assigned to.

### 4.2. Collaborative Monitoring

By merging the multiple sketches, we propose a mechanism of collaborative monitoring to improve the estimation accuracy of flows. We can make a flow with poor estimated accuracy be measured in an additional Count–Min sketch with low resource utilization. For the example shown in Figure 1b, suppose there are two flows $f_{b}$ and $f_{c}$, passing through monitor $m_{1}$ and monitor $m_{3}$ at the same time. In the beginning, $f_{b}$ is assigned to monitor $m_{1}$ and $f_{c}$ is assigned to monitor $m_{3}$. Now we assume that the resources of monitor $m_{3}$ are not fully utilized, then we can also assign flow $f_{b}$ to monitor $m_{3}$. Since the measurement load of monitor $m_{3}$ is small and the probability of the hash collision is low, the estimated result of $f_{b}$ on monitor $m_{3}$ is likely more accurate than that on monitor $m_{1}$. Thus, the estimated accuracy of the flow $f_{b}$ will be improved by merging the results of two monitors.

From Theorem 1, we can see that there are two problems with accuracy-aware collaborative measurements. The first problem is possibly overloaded monitors. The same flow may be assigned to multiple monitors, so the measurement load of each monitor will inevitably increase. In particular, when the network topology is irregular, the traffic through each monitor is non-uniform. For example, fat-tree topology is a typical and practical topology for data center networks. There are three layers, i.e., edge layer, aggregation layer, and core layer, respectively, from top to bottom. Obviously, the measurement load in the core layer is heaviest since a large number of flows converge in monitors in the core layer. Thus, it is likely to overwhelm the monitor to perform collaborative monitoring in the core layer.

When the load of a monitor is too heavy, the processing queue of the monitor becomes full and the later packets will be discarded directly, resulting in a decrease in measurement accuracy. Moreover, according to the error bound of the sketch in Equation (2), a large number of measured flows will lead to the expansion of the error range of the sketch. It is most likely that the estimated results from the monitors in the core layer are less accurate than those from the ingress monitors.

Thus, collaborative monitoring will increase the load of the monitors, and the measurement results will worsen.

While the second challenge is flow selection. Collaborative measurements will improve the measurement accuracy of the current flow and reduce the measurement accuracies of other flows. When the resources of monitors are not enough to advocate idle monitors to all flows, how to choose flows to perform collaborative measurements is a challenge. Large flows are more important in network-wide measurements. Thus, we utilized collaborative monitoring to measure large flows, which is termed accuracy-aware collaborative monitoring.

## 5. Algorithms of Accuracy-Aware Collaborative Monitoring

In this section, We discuss the problem of accuracy-aware monitoring (PANM); PANM was transformed into a load balance optimization problem, which is NP-hard. Thus, we propose an approximation algorithm, named the ‘improved longest processing time algorithm’ (iLPTA). We propose a two-stage online distribution algorithm (TODA).

### 5.1. Problem of Accuracy-Aware Network-Wide Measurements

The ultimate objective of network-wide measurements at the flow level is to provide accurate flow-level measurements to guide several network management applications. However, since there is no direct theoretical model to explain measurement accuracy, many literature studies have converted the problem of network-wide measurements into a load balance problem [4,7,13,14]. Similarly, the goal of problem accuracy-aware network-wide measurements (PANM) is to improve the measurement accuracies of large flows while slightly affecting the measurement accuracies of other flows. The measurement accuracies of flows can be improved by collaborative monitoring. Thus, we can achieve the goal of PANM by performing collaborative monitoring for large flows. As long as collaborative monitoring works well, the goal of PANM can be achieved to a certain degree. From the error bound of collaborative measurements, we can conclude that the bottleneck of collaborative measurement accuracy based on the Count–Min sketch lies in the monitor with the largest total measured flow size. Thus, we can transform the PANM into the problem of the load balance of the total measured flow sizes of the monitors. This not only improves the measurement accuracy but also avoids the overloaded monitors. Based on the above analysis, our solution involves collaborative monitoring of large flows. The basic idea is for small flows, only one monitor is assigned, and load balance is considered to allocate the monitor; but for large flows, multiple monitors are advocated, and the load balance is also considered to allocate flow monitor resources. Then we can transform PANM into the load balance optimization problem as follows:

(5) $$\begin{array}{cc} \min_{d_{ij}}\max_{i} & \;N(m_{i}) \end{array}$$

(6) $$\begin{array}{ccc} s.t.\;\; & N\left(m_{j}\right)=\sum_{i:f_{i}\in F}d_{ij}*a_{i} & \;\forall m_{j}\in M, \end{array}$$

(7) $$\begin{array}{ccc} \; & \sum_{j:m_{j}\in P_{f_{i}}}d_{ij}=1 & \;\forall f_{i}\in S, \end{array}$$

(8) $$\begin{array}{ccc} \; & \sum_{j:m_{j}\in P_{f_{i}}}d_{ij}\geq \beta & \;\forall f_{i}\in L, \end{array}$$

(9) $$\begin{array}{ccc} \; & \sum_{i:f_{i}\in F}d_{ij}\leq C_{m_{j}} & \;\forall m_{j}\in M, \end{array}$$

(10) $$\begin{array}{ccc} \; & d_{ij}\in \left\{0,1\right\} & \;\forall f_{i}\in F,\forall m_{j}\in M. \end{array}$$

$N(m_{j})$ denotes the total measured flow size on the monitor as Equation (6). We introduce a predefined threshold θ to classify the full flow set F into a small flow set S and a large flow set L based on the flow size. Our decision variable is $d_{ij}$, which denotes whether $f_{i}$ is measured on monitor $m_{j}$. The first constraint of $d_{ij}$ is that each flow from a small flow set should be measured once, Equation (7), while each flow from a large flow set should be measured at least β times, such as Equation (8). The fourth constraint is that each monitor $m_{j}$ should measure at most $C_{m_{i}}$ flows because of the existence of hash collisions, Equation (9). The last constraint of $d_{ij}$ is that the value of $d_{ij}$ is either equal to 0 or 1, such as in Equation (10).

It is obvious that the min–max problem is an integer linear programming problem. The branch and bound methods [26] are widely used to solve integer linear programming problems. Moreover, some extensive solvers have integrated this method, such as CPLEX [27] and Gurobi [28].

### 5.2. Approximation Algorithm

Since the min–max problem is an integer linear programming that is NP-hard, finding the optimal solution to the load-balance problem is intractable in large-scale networks. This subsection presents an approximation algorithm named the improved longest processing time algorithm (iLPTA), which is used for finding approximate solutions to the min–max problem efficiently.

In order to simplify the min–max problem, we conducted a simple experiment to show how the number of monitors to which large flows were distributed varied. We chose fat-tree topology containing 20 switches, and network traffic data contains 38,713 flows with a total size of 249,824. We set β to 2 and take the 3000 top-ranked flows as large flows. Next, we used Gurobi to solve the min–max problem. The result shows that all large flows are distributed to two monitors although large flows can be distributed to more than two monitors according to Equation (8). Thus, in the rest of this paper, we replaced Equation (8) with Equation (11) for convenience.

(11) $$\sum_{j:m_{j}\in P_{f_{i}}}d_{ij}=\beta \;\forall f_{i}\in L.$$

Because the min–max problem is a load-balance problem, we can convert our problem into a scheduling problem. First, we deleted Equation (9) to simplify the problem. This constraint was used to decrease hash collisions, not hard constraints. Second, we set β to 2, where large flows were measured on two monitors. Then the conversion from the min–max problem to scheduling problems was as follows: (1) each flow with a flow size $a_{i}$ was mapped to a job with the processing time $a_{i}$ and each monitor was mapped to a machine; (2) the objective can be regarded as the maximum load over all machines; (3) large flows were regarded as two jobs with same processing time with the constraint that they could not be allocated to the same machine; (4) each job was only allocated to its candidate machines corresponding to the routing path.

Using the three-field notation of Graham et al. [29], the above problem is denoted as $P||C_{max}$ problem with an additional constraint, where *P* represents the identical parallel machines environment and $C_{max}$ is the makespan objective function. Inspired by the longest processing time algorithm in [30], we present an improved longest processing time algorithm (iLPTA) because of the additional constraint. The difference is that each large flow cannot be distributed to two identical monitors.

The intuition behind Algorithm 1 is the following: we sorted the new Array containing small flows once and large flows twice based on the flow size of each item in descending order, then we distributed flow $f_{i}$ to monitor its routing path, whose load was the smallest. In order to not distribute large flows to two identical monitors, we allocated a monitor whose load was the second-smallest in line 11 when this case happened. The algorithm consisted of two layers of iteration with the first one traversing all flows, and the second one selecting the monitor whose load was the smallest. Thus, the complexity of iLPTA was O(nt). 

**Algorithm 1:** Improved longest processing time algorithm (iLPTA).

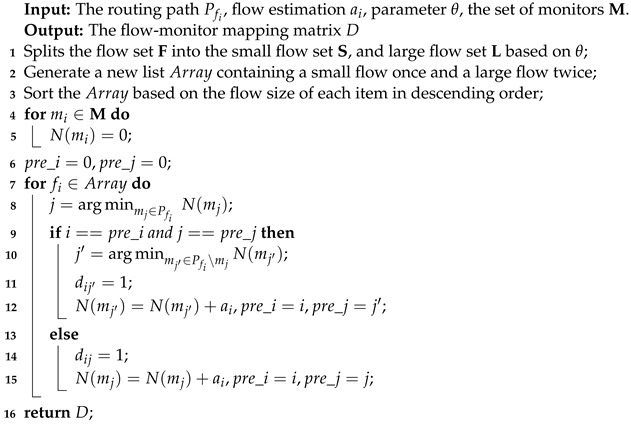



We prove the approximation ratio of iLPTA, where it is the ratio of the maximum load *W* produced by iLPTA divided by $W^{*}$ produced by the optimal solution.

**Lemma** **1.** The optimal load $W^{*}\geq \frac{\sum_{m_{k}\in M}N\left(m_{k}\right)}{n}$.

**Proof.**  The total measured flow size is $\sum_{m_{k}\in M}N\left(m_{k}\right)$, one of *n* monitors must do at least a $\frac{1}{n}$ fraction of the total work.    □

**Lemma** **2.** If there are more than n flows, $W^{*}\geq 2\cdot a_{n+1}$.

**Proof.**  Consider first n+1 flows $a_{1},a_{2}\cdots a_{n+1}$. Since the $a_{i}$ is in descending order, $a_{i}\geq a_{n+1}$, there are n+1 flows and *n* monitors, so by the pigeonhole principle, at least one monitor measures two flows.    □

**Theorem** **2.** *The iLPTA algorithm is a $\left(\frac{1}{2}+\frac{n}{l}\right)$ approximation algorithm, while m denotes the number of all monitors and $l=\min_{f_{i}\in F}\left|P_{f_{i}}\right|$.*

**Proof.**  Consider load $N(m_{i})$ of the bottleneck monitor $m_{i}$. Let $f_{j}$ be the last flow distributed on monitor $m_{i}$. Because the Array is sorted based on the flow size in descending order, $f_{j}$ is a small flow and will be distributed to only one monitor. When flow $f_{j}$ is distributed to monitor $m_{i}$, $m_{i}$ has the smallest load among all monitors on its routing path. The load before the assignment is $N\left(m_{i}\right)-a_{j}$. Thus, $N\left(m_{i}\right)-a_{j}\leq N\left(m_{k}\right)$ for all $m_{k}\in P_{f_{i}}$. Next, we sum the inequalities over $m_{k}$ and divide by $|P_{f_{i}}|$.

(12) $$\begin{array}{cc} N\left(m_{i}\right)-a_{j} & \leq \frac{\sum_{m_{k}\in P_{f_{i}}}N\left(m_{k}\right)}{|P_{f_{i}}|}=\frac{n}{|P_{f_{i}}|}\frac{\sum_{m_{k}\in P_{f_{i}}}N\left(m_{k}\right)}{n}\leq \frac{n}{|P_{f_{i}}|}\frac{\sum_{m_{k}\in M}N\left(m_{k}\right)}{n}\\ & \leq \frac{n}{|P_{f_{i}}|}W^{*}. \end{array}$$

Thus,

(13) $$\begin{array}{cc} N(m_{i}) & =\left(N\left(m_{i}\right)-a_{j}\right)+a_{j}\leq \frac{n}{|P_{f_{i}}|}W^{*}+\frac{W^{*}}{2}\\ & =\left(\frac{1}{2}+\frac{n}{|P_{f_{i}}|}\right)\cdot W^{*}=\left(\frac{1}{2}+\frac{n}{l}\right)\cdot W^{*}. \end{array}$$

   □

The solution of the min–max problem not only solves the problem of load balance but also improves the measurement accuracy to a certain extent. However, the flow size in the formula is not known in advance, and it is the final result of the network measurement. Thus, how to estimate the flow size becomes a challenge for the problem.

We can use the time window method and take the flow size of the last time window as one of the current time windows to solve the above min–max problem. The main reasons are as follows: the traffic flow follows the Zipf distribution; that is, the 20% of top-ranked flows account for more than 80% of total traffic. Thus, the large flow mainly dominates the load of a monitor. As a result, the flow size estimation should cover large flows. Large flow may last for multiple time windows due to the large flow size. This kind of method will not cause a large error.

### 5.3. Two-Stage Online Distribution Algorithm (TODA)

However, because network flows arrive at monitors asynchronously, the controller does not know which flow will arrive in the current time window in advance. Therefore, we propose a two-stage online distribution algorithm (TODA) to ensure load balance as much as possible.

In the first stage, the controller will assign a monitor with the lightest load for each new flow. Each monitor stores a threshold θ. When the estimated value of a certain flow exceeds the threshold θ, the monitor will report to the controller. Then this flow will enter the second stage. Another monitor is allocated for the current large flow for collaborative measurement, and the corresponding cells of this monitor are set to θ.

**Figure 5:**
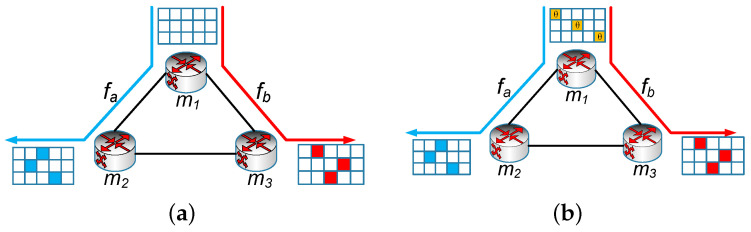
Two-stage online distribution algorithm (TODA). (**a**) First Stage; (**b**) Second Stage.

As shown in Figure 5, there are three monitors in the network. We focus on a small flow $f_{a}$ and a large flow $f_{b}$ in the network. In the first stage, when flow $f_{a}$ and flow $f_{b}$ enter the network, the controller assigns monitor $m_{2}$ and monitor $m_{3}$, respectively, as shown in Figure 5a. When the time goes, the number of corresponding cells onto which flow $f_{a}$ and flow $f_{b}$ are mapped increases. For large flow $f_{b}$, when its estimated value on monitor $m_{3}$ exceeds the threshold θ, then it will enter the second phase. The controller will allocate another monitor $m_{1}$ for flow $f_{b}$, and set the value of the corresponding cells of monitor $m_{1}$ to θ, while small flows will stay in stage one because the flow size is small, as shown in Figure 5b.

We provide the pseudo-code of the TODA in Algorithm 2. As for each small flow, we allocate only a monitor with the smallest total measured flow size on its routing path when entering the network in lines 3–4. However, for each large flow, we allocate a new monitor when its estimation reaches θ in lines 8–9.

**Algorithm 2:** Two-stage online distribution algorithm (TODA).

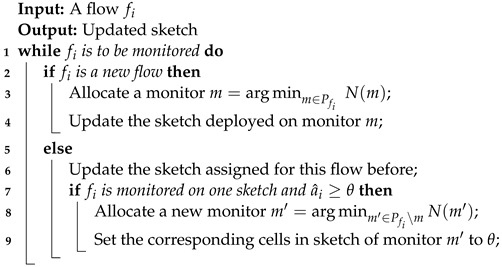



The online algorithm is very similar to the list-scheduling algorithm where each job is assigned to a machine whose load is the smallest so far, considering *t* jobs in some fixed order. Now, we prove the approximation ratio of the TODA algorithm, where it is the ratio of the maximum load *W* produced by the online algorithm divided by $W^{*}$ produced by the optimal solution.

**Lemma** **3.** The optimal load $W^{*}\geq \max_{f_{i}\in F}a_{i}$.

**Proof.**  Some monitors must measure the flow with the largest flow size.    □

**Theorem** **3.** *The TODA algorithm is a $\left(1+\frac{n}{l-1}\right)$ approximation algorithm, while m denotes the number of all monitors and $l=\min_{f_{i}\in F}\left|P_{f_{i}}\right|$.*

**Proof.**  Consider load $N(m_{i})$ of the bottleneck monitor $m_{i}$. Let $f_{j}$ be the last flow distributed on monitor $m_{i}$. Because we do not know the flow size in advance, it is possible that $f_{j}$ is a large flow. Then $f_{j}$ will be distributed to a monitor, except the first one for this flow. Let $m_{q}$ be the first monitor to measure $f_{j}$. When the flow $f_{j}$ is distributed to monitor $m_{i}$, $m_{i}$ has the smallest load among all monitors except $m_{q}$ on its routing path. Its load before the assignment is $N\left(m_{i}\right)-a_{j}$. Thus, $N\left(m_{i}\right)-a_{j}\leq N\left(m_{k}\right)$ holds for all $m_{k}\in P_{f_{i}}\setminus m_{q}$. Next, we sum inequalities over all $m_{k}$ and divide by $|P_{f_{i}}|-1$.

(14) $$\begin{array}{cc} N\left(m_{i}\right)-a_{j} & \leq \frac{\sum_{m_{k}\in P_{f_{i}}\setminus m_{q}}N\left(m_{k}\right)}{|P_{f_{i}}|-1}\\ & =\frac{n}{|P_{f_{i}}|-1}\frac{\sum_{m_{k}\in P_{f_{i}}\setminus m_{q}}N\left(m_{k}\right)}{n}\\ & \leq \frac{n}{|P_{f_{i}}|-1}\frac{\sum_{m_{k}\in M}N\left(m_{k}\right)}{n}\\ & \leq \frac{n}{|P_{f_{i}}|-1}W^{*}. \end{array}$$

According to Lemma 3, we can deduce the following:

(15) $$\begin{array}{cc} N(m_{i}) & =\left(N\left(m_{i}\right)-a_{j}\right)+a_{j}\\ & \leq \frac{n}{|P_{f_{i}}|-1}W^{*}+W^{*}\\ & =\left(1+\frac{n}{|P_{f_{i}}|-1}\right)\cdot W^{*}\\ & =\left(1+\frac{n}{l-1}\right)\cdot W^{*}. \end{array}$$

   □

### 5.4. Discussion

The proposed approximation algorithm (iLPTA) and the online algorithm (TODA) have similarities and differences. (1) The similarity is that both are adopted to choose monitors for flows. (2) The difference is that iLPTA is an offline approximation algorithm that not only solves the problem of load balance but also improves the measurement accuracy to a certain extent. The key idea of iLPTA is to allocate more monitors to large flows and achieve load balance to provide accuracy-aware monitoring. However, the flow size in the min–max problem is not known in advance, and it is the final result of the network measurement. Thus, how to estimate the flow size becomes a challenge for the problem. We propose TODA, an online algorithm that attempts to achieve the load balance by selecting a monitor with the smallest load to a large flow. The offline iLPTA algorithm proves the validity and feasibility of the core idea, and the TODA is the online algorithm utilized in practice.

## 6. Experiments

In this section, we first introduce the metrics and benchmarks for the performance comparison. We evaluate our proposed iLPTA and TODA by comparing them with some benchmarks through extensive experiments.

### 6.1. Performance Metrics and Benchmarks

In this paper, we designed a mechanism of accuracy-aware collaborative monitoring for network-wide measurements to improve the estimation accuracies of large flows. Thus, we used the following metrics in our numerical evaluations: (1) Maximum measurement packets: the maximum measurement packets among all monitors; (2) maximum measurement flows: the maximum measurement flows among all monitors; (3) ARE (average relative error): the average relative error was calculated by $\frac{1}{t}\sum_{i=1}^{t}\frac{\left({\hat{a}}_{i}-a_{i}\right)}{a_{i}}$; (4) AAE (average absolute error): the average absolute error was calculated by $\frac{1}{t}\sum_{i=1}^{t}\left({\hat{a}}_{i}-a_{i}\right)$. At the end of an epoch, we could determine the number of packets measured by each monitor, and use the maximum one as the first metric. The number of flows measured by each monitor could also be determined; we chose the maximum one as the second metric. For the third and fourth metrics, we queried all of the flows from the Count–Min sketches and calculated AAE and ARE. We only had to query one Count–Min sketch to estimate the small flows and two sketches for the large flows. Because collaborative monitoring could improve the accuracies of large flows while harming one of the small flows, we calculated not only the overall AAE and ARE for all flows but also the counterparts for large flows.

Because solving the min–max problem is really time-consuming, we could not obtain the optimal solution. We just evaluated the performance of our proposed iLPTA and TODA and compared them with three other different algorithms with the objectives mentioned above: (1) **NSPA** [7]: all flows were distributed to monitors uniformly; (2) **Random**: each flow was distributed to a monitor within the routing path randomly; (3) **IO**: each flow was measured only by its ingress monitor.

### 6.2. Experiment Settings

In the experiment, we selected a typical and practical topology for data center networks, fat-tree topology. It contained 20 switches (including 8 edge switches, 8 aggregation switches, and 4 core switches) and 16 terminals. We assumed that the terminals were also switches and deployed one Count–Min sketch at every switch. The width *w* of each sketch changed from 500 to 1500, and the height *d* of each was equal to 3. For iLPTA, we took the 3000 top-ranked flows, which accounted for eighty percent of total traffic as large flows. However, for TODA, we set θ as 100, which was larger than that of iLPTA. This is because the estimation of the Count–Min sketch is always overestimated. Thus, a larger θ can ensure that small flows will not be monitored twice, but the measured number of large flows will be less than 3000. We used four one-hour public traffic traces collected in Equinix Chicago monitor from CAIDA [31]. We divided each trace into different time intervals (1 s, 5 s, 10 s, 30 s, and 60 s) and evenly partitioned the traces and distributed them across hosts. We used the CAIDA4 trace with a monitoring time interval of 5 s as the default trace, which contained 1.1 M to 2.8 M packets with flows of 30 K to 50 K (SrcIP). Due to space limitations, we only show the results with the source IP as the flow ID; the results are qualitatively similar for other flow IDs (e.g., destination IP, 5-tuple).

### 6.3. Impact of Sketch Size

**Figure 6:**
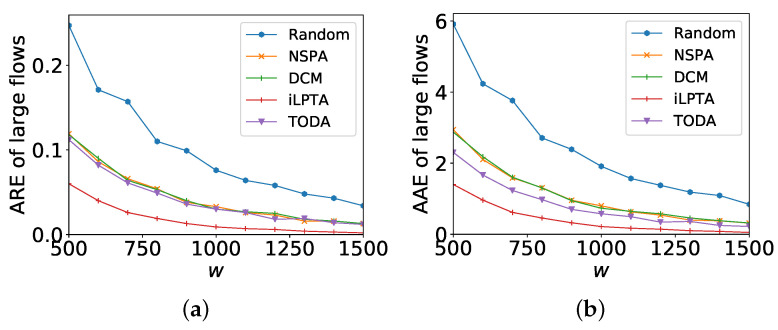
Measurement accuracies of large flows with different *w*. (**a**) ARE; (**b**) AAE.

Figure 6 shows the measurement accuracies of large flows with different *w*. AAE and ARE of large flows by IO are the worst because all flows are measured on ingress monitors causing high hash collisions. Its performance is at least 4× worse than that of Random for the objectives of both AAE and ARE. Thus, we do not show the performance of IO in Figure 6. The width of each sketch increases; however, the measurement accuracies of all five algorithms decrease. For the ARE of large flows, iLPTA achieves the best results and at least 2× lower ARE of large flows than that of NSPA. When the width is set to 1500, the measurement accuracy can be improved by a factor of up to 6× compared to NSPA. Moreover, TODA achieves low ARE (close to that of NSPA). For the AAE of large flow, there are similar results. iLPTA also has the best result and at least 2× lower AAE of large flows than NSPA. Moreover, TODA achieves lower AAE than that of NSPA in most cases.

**Figure 7:**
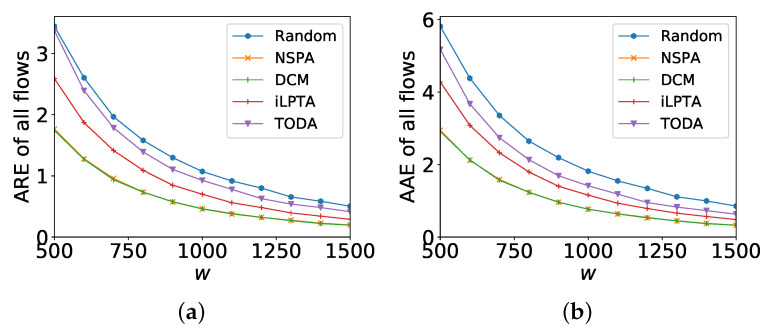
Measurement accuracies of all flows with different *w*. (**a**) ARE; (**b**) AAE.

Figure 7 shows the measurement accuracies of all flows with different *w*s. AAE and ARE of large flows by IO are the worst (at least 4× worse than that of random) and we delete the statistics by IO. The measurement accuracy by random is only better than that of IO. For the ARE of all flows, NSPA achieves the best result and averages 1.5× and 2.1× lower ARE of all flows than those of iLPTA and TODA, respectively. Even when the width is set to 1700, the measurement accuracy by iLPTA is only worse by a factor up to 1.5× compared to NSPA. For the AAE of large flows, there are similar results. NSPA achieves the best result and averages 1.5× and 2.2× lower ARE of all flows than that of iLPTA and TODA, respectively.

From Figure 6 and Figure 7, we can conclude that iLPTA always works better than TODA because of the awareness of the flow size, and iLPTA and TODA can improve the measurement accuracies of large flows (e.g., at least 2× lower AAE and ARE and slightly affect the ARE or AAE of all flows (e.g., average 1.5× higher AAE and ARE).

### 6.4. Impact of Fraction of Large Flows

**Figure 8:**
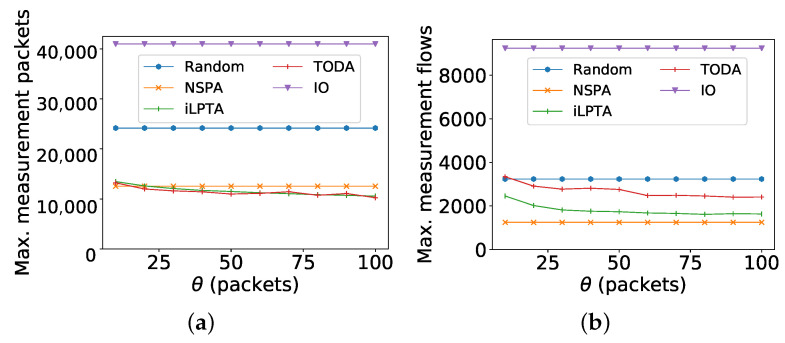
Impact of the fraction of large flows with different θ. (**a**) Maximum measurement packets; (**b**) Maximum measurement flows.

One important parameter in our experiments is θ, which is used to classify large flows and small flows. We show the impact of large flow fractions with different θ in Figure 8. In all experiments, θ changes from 10 to 100, and *w* is set as 1000. Figure 8a,b show the objectives of maximum measurement packets and maximum measurement flows, respectively. First of all, these two metrics by three comparison algorithms (IO, Random, and NSPA) remain unchanged. This is because the performances of the three algorithms have nothing to do with the parameter θ. Secondly, while the parameter θ increases, the two metrics by both iLPTA and TODA decline because fewer flows and less packets are measured with a higher parameter θ. Finally, in Figure 8b, the maximum measurement flow by NSPA is the lowest because it distributes flows uniformly among monitors. Moreover, the maximum measurement flows by iLPTA and TODA are lower than those by random and IO. However, in Figure 8a, iLPTA and TODA achieve the lowest maximum measurement packets in most cases though more packets are measured by the two algorithms. From Figure 8, we can conclude that iLPTA and TODA can achieve load balances at the level of packets, which is vital to sketches better than NSPA in most cases.

**Figure 9:**
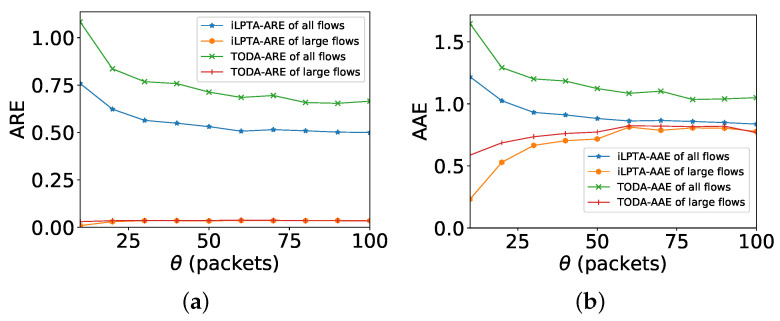
Measurement accuracy with different θ. (**a**) ARE; (**b**) AAE.

**Figure 10:**
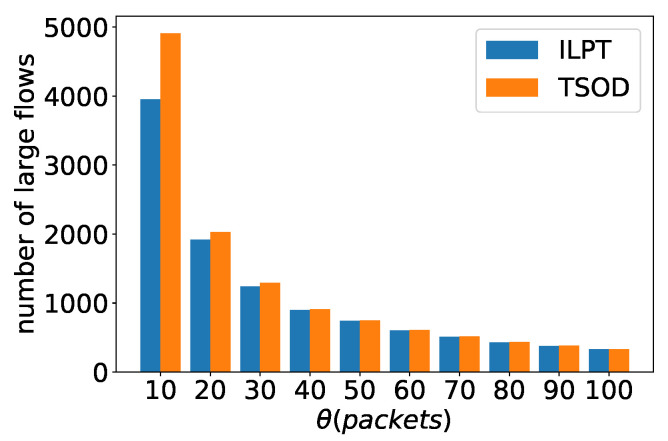
Number of large flows with different θ.

Figure 9a shows the objective of ARE with different θ. No matter how θ changes, the AREs of large flows by both algorithms are always better than those of all flows, and the ARE gap between large flows and all flows is very obvious. Thus, iLPTA and TODA can achieve lower AREs of large flows. Figure 9b shows the objective of AAE with different θ. Moreover, when θ increases, the AAEs of large flows by both algorithms are better than those of all flows. There are downward trends in the AAEs of all flows by both algorithms, while the value of θ increases. This means a small θ will harm the AAEs of other flows. The AAEs of large flows by two algorithms first increase when the parameter θ is less than a certain value, and then the AAEs decrease. Figure 10 depicts the number of large flows with different θ. The value of θ increases; however, the number of large flows decreases dramatically. Thus, a larger θ may not ensure that all large flows are measured.

From the above trends, it can be seen that when the threshold θ is relatively large, the large flow set is small. Although the measurement accuracy of the large flow is improved, it is difficult to cover all large flows. When the threshold θ is moderate, large flows can be well covered. The measurement accuracy of the large flow is improved, and there is a small impact on the overall measurement accuracy. However, when the threshold θ is small, the large flow set is large, and a part of the small flow is divided into the large flow. The measurement load of the network increases a lot, which decreases the overall measurement accuracy. At the same time, the promotion of the accuracy on the top-ranked flow is not very obvious. Therefore, the selection of the threshold θ should be based on the specific network environment.

## 7. Conclusions

In this paper, we studied the problem of flow distribution in network-wide software-defined measurements, considering final measurement accuracies and the differences between large flows and small flows. We proposed a mechanism of accuracy-aware collaborative monitoring (ACM) for network-wide measurements. Then we formulated the problem as an integer linear programming problem, which is NP-hard. Thus, we proposed an approximation algorithm named iLPTA and proved that its approximation ratio is $\left(\frac{1}{2}+\frac{n}{l}\right)$. We provide an online algorithm for the problem. The online algorithm does not require any knowledge about the flow size. Through extensive experiments, we showed that our iLPTA and TODA can improve the measurement accuracies of large flows while slightly affecting the overall accuracy. The experiments also show that our TODA can perform close to iLPTA. This paper only focuses on the flow-level network-wide measurements based on the Count–Min sketch; we will investigate superior measurement applications in future work.

## Figures and Tables

**Table 1:** Notations.

Notation	Description
$f_{i}\in F$	*i*th data flow
$m_{i}\in M$	*i*th monitor
θ	Threshold to distinguish large flow and small flow
S/L	Small flow set/Large flow set
n/t	Number of monitors / Number of flows
$a_{i}$, ${\hat{a}}_{i}$	Actual and estimated flow size
*D*	Flow-monitor mapping matrix
$d_{ij}$	Whether flow $f_{i}$ is measured on $m_{j}$
$N(m_{i})$	Total measured flow size on monitor $m_{i}$
$C_{m_{i}}$	Upper bound of the number of measured flows at monitor $m_{i}$
β	Lower bound of the number of monitors
$P_{f_{i}}$	Routing path of $f_{i}$
*l*	Minimum number of monitors all flows pass through
w,h	Width and height of Count–Min sketch

## Data Availability

The data presented in this study are available upon request from the first author.
